# Looking at the dark side: A bibliometric study on uncited and poorly cited articles in orthodontic research

**DOI:** 10.4317/jced.61962

**Published:** 2024-10-01

**Authors:** Daniele Garcovich, Erica Lipani, Pilar España-Pamplona, Riccardo Aiuto, Alfonso Alvarado-Lorenzo, Milagros Adobes-Martin

**Affiliations:** 1 Department of Orthodontics, European University of Valencia. Valencia, Spain; 2 Department Orthodontics, Universitá di Cagliari. Cagliari, Italy; 3 Department Orthodontics, European University of Valencia. Valencia, Spain; 4 Department of Biomedical Sciences, University of Milan. Milano, Italy; 5 Department Orthodontics, Universidad de Salamanca. Salamanca, Spain; 6 Department of Orthodontics, European University of Valencia. Valencia, Spain

## Abstract

**Background:**

Many studies have analysed the bibliometric characteristics of highly cited articles in dentistry, and orthodontics. However, scant attention has been paid to articles with low citation rates. The aim of this study is to identify author- and article-specific factors that may be associated with a low citation rate at least 6 years after publication.

**Material and Methods:**

In June 2023, a cross-sectional study was conducted on articles published between 2009 and 2018 in eight orthodontic journals indexed in the Journal Citation Report. The study recorded author- and article-specific variables for articles that received zero citations and those that received between one and three citations. Descriptive statistics were used to describe the articles and journals included in the study. Pearson’s correlation analysis was used to test the correlation between a journal’s impact factor and the number of low-cited articles for the individual journals. The articles’ related topics were further analysed using VOSviewer 1.6.6 software.

**Results:**

The electronic search identified 11,257 published items. After applying the inclusion and exclusion criteria, 216 uncited and 683 poorly cited articles were included in the final assessment. The Australasian Orthodontic Journal had the highest number of uncited and poorly cited articles, followed by Seminars in Orthodontics. A high negative correlation was found between the journal impact factor and the normalized number of uncited and poorly cited articles. The majority of uncited articles were expert opinions (28.24%), case reports (21.76%), and narrative reviews (21.30%). The most frequent topics were legislation, litigation, and ethics, followed by marketing and management. Most of the poorly cited articles were observational (29.43%) or translational studies (26.21%), and case reports (22.55%). The most prevalent topic in this cohort was eruption problems.

**Conclusions:**

The impact factor of a journal is linked to the number of published articles that receive a low citation rate. Orthodontics has a higher prevalence of such articles compared to other branches of medicine. Topics such as litigation, legislation, ethics, and marketing tend to receive low citation rates. Uncited articles often consist of expert opinions or narrative reviews. Case reports are a common study design in both uncited and poorly cited articles.

** Key words:**Bibliometric, Orthodontics, citations, Uncited.

## Introduction

Since the introduction of the first journal impact metric, the Journal Impact Factor (JIF), in 1965 by Eugene Garfield, founder of the Institute for Scientific Information (ISI), citations have been used as a surrogate measure of the quality of research. Citation metrics have been employed to evaluate the contribution and scientific performance of individual scientists, research groups, departments, and universities. Hiring, promotion, funding, and award selection have largely been decided thus far, mainly based on these data (1). Despite their widespread use, citation-based metrics are not free of criticism. The Declaration on Research Assessment (DORA) released in San Francisco, back in 2012, raised awareness of the perils of JIF-driven decisions. Initially, published by a small group of scientists and editors convened at the American Society for Cell Biology, the Declaration has since been signed by more than 16,544 individuals and some 2076 organisations and institutions (2).

The JIF is an index proposed by a commercial enterprise without prior consultation with the affected communities of scholars. The index was conceived as a tool to help librarians identify which journals to purchase and was not intended as a measure of the scientific quality of research (3). Furthermore, citation-based metrics have several limitations, such as the following: they do not consider the reason behind the article citation; they can be dramatically manipulated by editorial policies and self-citation practices; there is a time delay between the citation in a published article and the indexing in the citation database; and the so-called Matthew effect can alter citation behaviour, as the most cited papers are more likely to be cited even more.

Citation-based metrics need to be reconsidered, and currently, funders and universities are trying to implement new metrics that can theoretically recognise and reward citizenship, including community engagement, teamwork, and the emotional and practical labour that goes into research and that constitutes its societal impact (4).

Since classic citations are considered to be an indicator of research quality and impact, what are the features of poorly cited articles? Many studies have explored the bibliometric features of the most cited articles in medicine, dentistry and orthodontics, but little interest has been given to the least cited articles. The present study aimed to identify author- and article-specific data that might be associated with a low citation rate at least 6 years after publication.

## Material and Methods

This study did not constitute human subject research and therefore did not require approval from our institutional review boards.

In June 2023, a retrospective cross-sectional study was conducted on the items published between 2009 and 2018 in eight journals with a specific focus on orthodontics indexed in the Journal Citation Report (JCR) for the year 2019. These journals include the European Journal of Orthodontics (EJO), the American Journal of Orthodontics and Dentofacial Orthopedics (AJODO), the Angle Orthodontist (AO), the Korean Journal of Orthodontics (KJO), Orthodontics & Craniofacial Research (OCR), the Journal of Orofacial Orthopedics (JOO), the Australian Orthodontic Journal (AOJ), Seminars in Orthodontics (SO) and Progress in Orthodontics (PO). Moreover, due to the high interest of the orthodontic community in the items published in the Journal of Orthodontics (JO), this journal was also included in the search.

The articles were retrieved through a search in the Scopus database (https://www.scopus.com) using the following search string: ( SRCTITLE ( american AND journal AND of AND orthodontics AND dentofacial AND orthopedics ) OR SRCTITLE ( australian AND orthodontic AND journal ) OR SRCTITLE ( european AND journal AND of AND orthodontics ) OR SRCTITLE ( journal AND of AND orofacial AND orthopedics ) OR SRCTITLE ( korean AND journal AND of AND orthodontics ) OR SRCTITLE ( orthodontics AND craniofacial AND research ) OR SRCTITLE ( progress AND in AND orthodontics ) OR SRCTITLE ( seminars AND in AND orthodontics ) OR SRCTITLE ( the AND angle AND orthodontist ) OR SRCTITLE ( journal AND of AND orthodontics ) ) AND PUBYEAR > 2008 AND PUBYEAR < 2019 and selected according to the following inclusion and exclusion criteria.

The inclusion criteria were as follows: a) articles published by the nine journals included in the study; b) publication type (articles or reviews); c) articles published between 2009 and 2018; and d) items cited between 0 and 3 times.

The exclusion criteria were as follows: a) letters, obituaries, notes, short surveys, editorials and commentaries; b) meeting abstracts; c) conference papers; d) articles without recorded authors; e) retracted articles; and f) items that received more than 3 citations.

For the selected items, the following data were recorded on an Excel datasheet (Microsoft Office for Mac 2011 package): (a) article title; (b) journal title; (c) year of publication; (d) number of authors and affiliations; (e) authors’ gender; (f) type of affiliation of the authors, university or other; (g) article subject; (h) study type; (i) number of citations; l) open access or subscription based; and m) funded or not funded. The country/region of origin, as defined by the authors´ institutional affiliations, was retrieved by means of the Data Fetcher App version 7.4.3 powered by Scopus. The IF and the Scimago Journal Rank (SJR) were recorded if available for each journal for each year included in the search.

The retrieved items were further classified as uncited (0 citations) or poorly cited (1 to 3 citations). If the study design was not stated in the title or abstract, the full text was analysed, and the study type was identified according to the categories reported by Farjo *et al*. (2015). The articles were stratified by topic using the categories presented in previous bibliometric studies published in the field of orthodontics (5,6).

The articles’ related topics were further analysed using VOSviewer 1.6.6 software, which constructs and visualises bibliometric networks (http://www.vosviewer.com/, Leiden University Centre for Science and Technology Studies). The science mapping of articles was performed at all keyword co-occurrence levels.

Descriptive statistics using counts and proportions were employed to describe the articles and journals included in the study. Pearson’s correlation analysis was used to test the relationship between SJR and the number of uncited and poorly cited articles for the individual journals.

## Results

-Uncited articles

**Figure 1:**
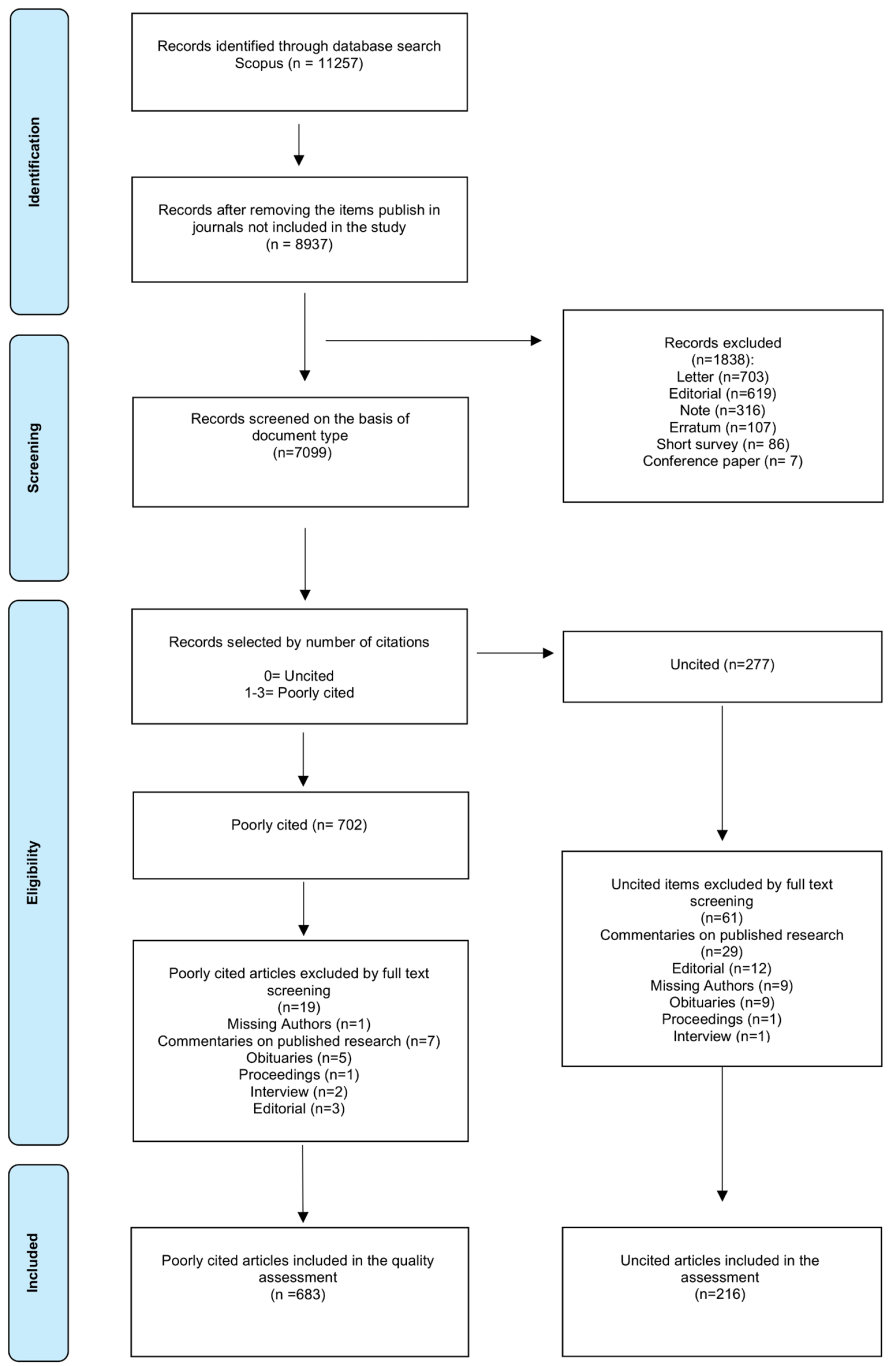
Flow diagram of the performed search.

**Figure 2:**
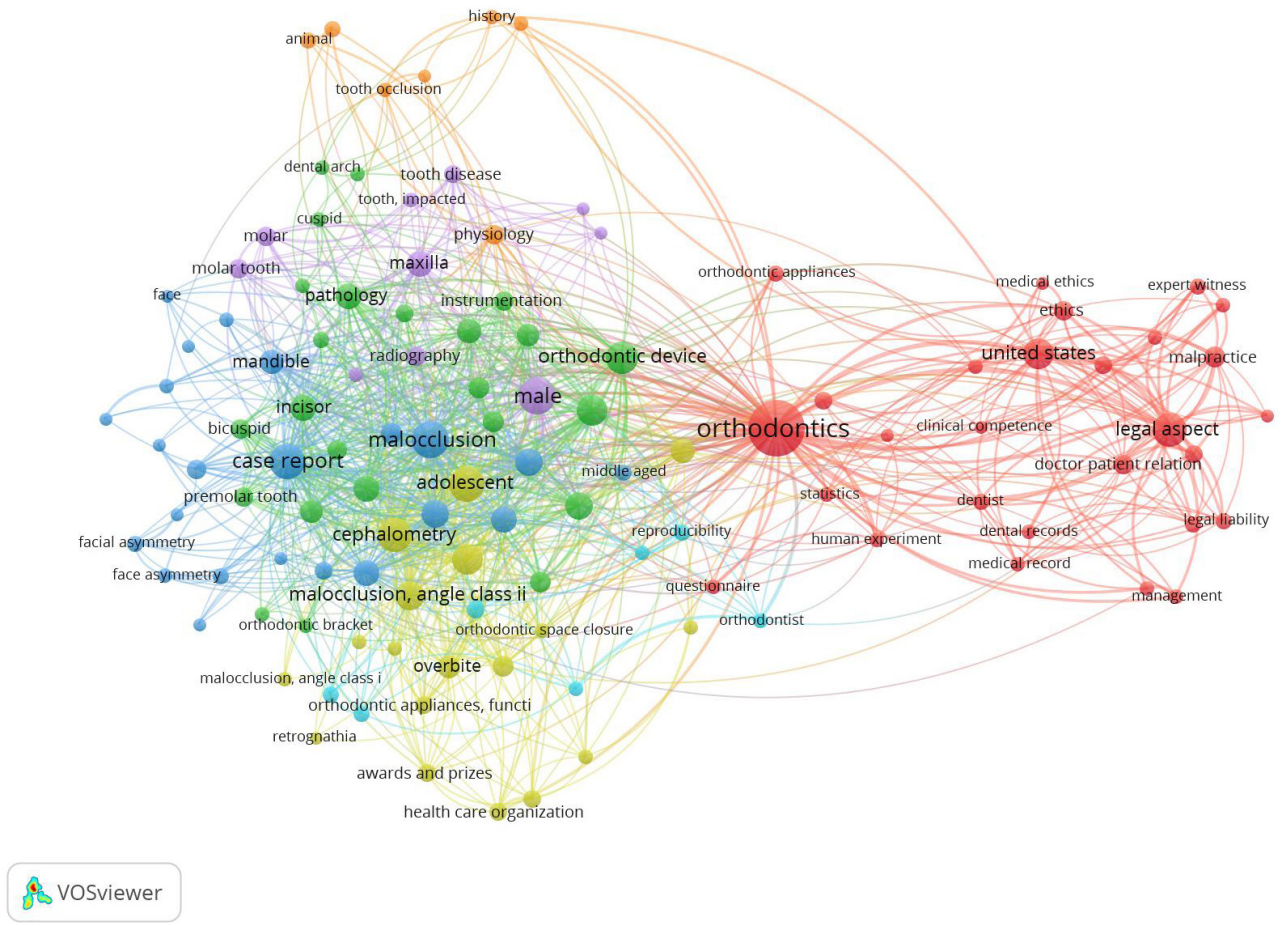
Co-occurrence keyword mapping of the uncited articles. The keywords belonging to the same cluster are shown in the same colour.

The electronic search identified 11257 published items; after applying the inclusion and exclusion criteria, 277 uncited articles were selected and assessed for eligibility; after screening the full text, 61 items were excluded at this stage, and 216 were included in the final assessment. The flowchart of the screening process is shown in Figure 1. Uncited articles represented 3.04% of the 7099 articles published by the selected journals during the studied period ( Table 1). Looking at absolute values, the journal that published the greatest number of uncited articles was AJODO, but it also published the greatest number of articles; normalising the number of uncited articles by the number of articles published by each journal, AOJ, followed by Seminars in Orthodontics, had the greatest number of uncited articles. The correlation between SJR and the normalised number of uncited articles was strong and negative (rs= -0.806, *p*= 0.00486). The number of uncited articles was not constant throughout the study period, with a peak in 2011. According to the study design, the vast majority were expert opinions (28.24%), case reports (21.76%) and narrative reviews (21.30%) ( Table 2). When the articles were stratified by topics, Legislation litigation and ethics accounted for 24.54% of the articles, followed by Marketing and management (10.19%) ( Table 3). Scientific mapping via keyword occurrence highlighted the existence of two distinct clusters, one related to purely orthodontic topics and another related to Legal, Marketing and management topics; the size of this cluster highlights its high prevalence (Fig. 2). As reported in  Table 4, most of these articles were written by a single author (52.31%), while only 12.50% involved five or more authors; the average number of authors per article was 2.31 ±1.78. The ratio of male to female authors was 2.64. The articles involved 202 institutions, 25 of which were non-academic. Non-academic institutions were involved in 62 of the published items, with Orthodontic Consulting Group being involved in 28 of them and being the most prevalent affiliation. Jerrold, L. authored 42 of the poorly cited articles and was the most frequent author. Of the 216 published items, 210 received no financial support, while 6 were funded by a total of 9 institutions. A total of 196 items (90.74%) had subscription-based access, while 20 (9.26%) had open access. Regarding the geographic origin of the authors, most of the articles were authored by North American researchers (39.61%), followed by European (34.18%) and Asian (13.78%) researchers.

-Poorly cited articles

**Figure 3:**
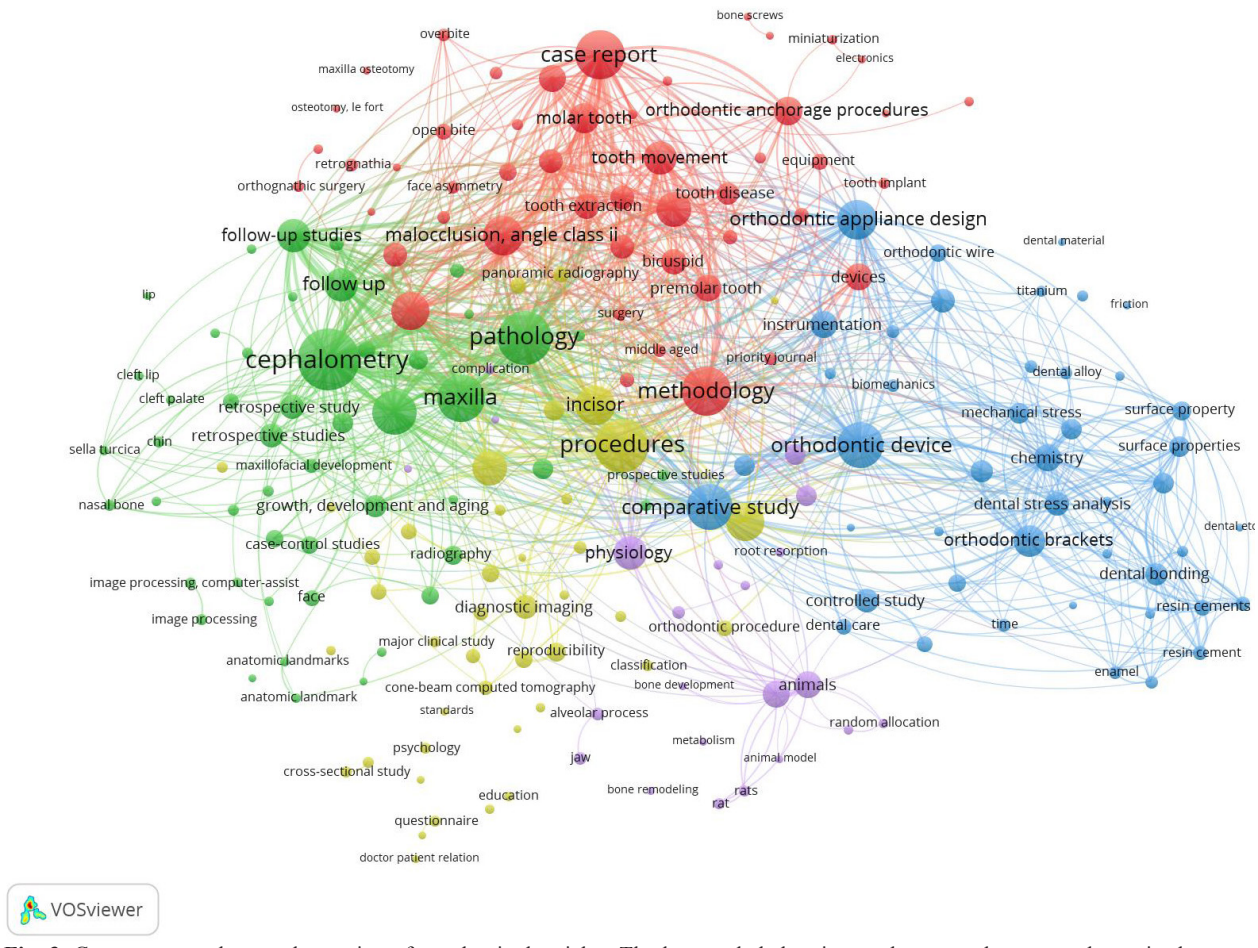
Co-occurrence keyword mapping of poorly cited articles. The keywords belonging to the same cluster are shown in the same colour.

The electronic search identified 11257 published items during the study period; 702 poorly cited articles were selected after applying the inclusion and exclusion criteria, and 19 were excluded after the full-text screening. A final pool of 683 articles was included in the final assessment. The flow chart of the screening process reporting the reasons for exclusion is displayed in Figure 1. As shown in  Table 5, the poorly cited articles represented 9.69% of the 7099 articles published by the selected journals during the study period. In absolute terms, the journal that published the greatest number of poorly cited articles was the AJODO, but when the number of poorly cited articles was normalised by the number of articles published by each journal, the greatest number of uncited articles was published in the AOJ (26.27%), followed by Seminars in Orthodontics (26.09%). The correlation between the SJR and the normalised number of uncited articles was strong and negative (rs= -0.891, *p*= 0.00054). The number of uncited articles displayed an almost constant pattern until 2015 but peaked in 2017-2018, ranging from 53 to 87 items per year over the study period. When the articles were stratified by study design, the most prevalent were observational (29.43%), translational (26.21%) and case reports (22.55%). Narrative review was also a prevalent study design, accounting for 13.91% of the published items ( Table 2). In terms of article topic, the most prevalent was Eruption problems: impaction, canine ectopic eruption/number problems (supernumeraries and agenesis) (6.44%), followed by Therapeutics, techniques, and procedures in second phase of treatment (6.30%), and Bone anchorage: Implants and screws (5.86%) ( Table 3). As highlighted in Figure 3, scientific mapping via keyword co-occurrence highlights the presence of three main clusters, one centred on the keyword cephalometry, another mainly centred on orthodontic device and orthodontic appliance design, and a third centred on case reports and methodology. As shown in  Table 4, 512 institutions were involved in the articles, 46 of which were non-academic. With 21 articles, Seoul National University was the most prevalent institution, followed by Kyung Hee University with 15 published items. Cho, J.H. was the most frequent author, with 10 published articles, followed by Baek, S.H. and Jerrold, L., with 9 articles. Of the 683 poorly cited articles, 616 did not receive financial support, while 67 items were funded by a total of 49 institutions. A total of 33.82% were Open Access. Regarding the geographic origin of the authors, most of the articles were authored by European researchers (32.42%), followed by Asian (29.70%) and North American (26.54%) researchers.

## Discussion

The impact factor of a journal is calculated by dividing the number of citations received by the journal in the last two years by the number of citable items. As suggested by previous studies on this topic, letters, obituaries, editorials, and commentaries were excluded from the search. The decision was made because although they may attract reader interest, they are non-ciTable items that are not included in the IF calculation(7). The chosen time frame of at least 5 years is supported by evidence in the bibliometric literature, which suggests that articles in the orthodontic field require 5 to 10 years to fully express their citation potential(8). The citation window (1 to 3) used to classify the low-cited items was chosen according to what was previously reported in other fields of medicine. Mullins *et al*. (2019) determined a citation window from 1 to 5 in the field of general surgery(9), Boyd *et al*. (2018) used a 0 to 1 citation span to classify the lowest cited articles in the field of urology (10), Warren *et al*. (2019) in the field of maxillofacial surgery defined as low cited, articles that received 0 to 3 citations, at least 5 years from publication (11), while Ranasinghe *et al*. (2015) used a cut-off value of five citations in cardiovascular journals (12). In medicine, the citation rate is greater than that in dentistry; for example, in the category “Urology and Nephrology”, a JCR category similar in size to “Dentistry Oral Surgery and Medicine”, the highest impact factor was 20.711, while in dentistry, it was 7.718. Due to the difference in the citation rate among the different fields, we decided to adopt the lower citation intervals among those reported. The high and negative correlation between the prevalence of uncited or poorly cited articles and the IF of a journal is consistent with what has been reported by other authors in other fields of medicine (9-11) and suggests that a higher impact factor is related to more stringent review processes that allow for the selection of significant articles that will contribute to the development of future research in the field. The Scopus database was preferred over the Science Citation Index Expanded of the Web of Science (SCI) for the article search in this study. This is because some of the journals such as Progress in Orthodontics, Orthodontics and craniofacial research and Seminars in Orthodontics, were not fully indexed in this database during the studied period. Additionally, previous research has shown a strong correlation between the number of citations in each database. The results of the search in the Scopus database revealed that the AOJ was not properly indexed in the database between 2017 and 2018, which can be considered a weakness of the study. However, a comparative search that we did on WOS highlighted that this journal was not indexed in this database in 2018 and was only partially indexed in 2017. Garfield, in a series of articles about uncitedness, reports that there are two main reasons for being uncited: first, in his own words, is “the uncitedness of the mediocre, the unintelligible, the irrelevant, the eccentric which leads to Index Oblivion” and second the one of the “meritorious or forgotten, the uncitedness of the village Milton of the scientific research” (13). According to his statements, uncitedness is ligated to something negative, but it should be considered that citations are not able to explore the full impact of a published item, as highlighted by the rise in the last decade of alternative metrics and altmetric aggregators (14). Pendlebury, D.A. in contrast to Garfield, argued that “certain level of non-citation in a journal is probably more an expression of the process of creation and dissemination of knowledge than any kind of performance measure”. Many authors have analysed the most cited articles in almost all fields of medicine and dentistry. In the orthodontic field, such bibliometric analyses have been performed several times (15), but to the best of our knowledge, uncited or poorly cited articles have never been analysed in the field of orthodontics. In our sample, we retrieved 216 uncited articles; this number is greater than what has been reported in other fields of medicine. Rosenkrantz *et al*., in 2018, retrieved only 47 uncited articles from a sample of 13,450 articles published over a decade in the field of radiology (16). Boyd *et al*. highlighted a similar number of 50 uncited articles after examining the four major journals in the field of urology (10). Regarding poorly cited articles, we retrieved 683 articles ranging from 1 to 3 citations. In 2018, Mullins *et al*. reported that only 50 articles with 0-5 citations were published by three major general surgery journals over a decade, while in maxillofacial surgery, only 4 articles with 0 citations and 62 articles with 1-3 citations were identified during the 10-year period after publication in four major journals (11). On the other hand, in 2017, Belloni Cuenca *et al*. reported a high rate of uncitedness, ranging from 5.7% to 58.1%, when studying eight Brazilian public health journals over a four-year period; however, their results cannot be easily compared to ours because the explored journals were indexed in Scopus but did not have the highest impact factor in their field, as did our sample (17). Moreover, some of the included journals pertain to the field of humanities in public health; according to some authors, the humanities journals analysed in the Web of Science database can reach up to 98% of uncited article rates, being the highest in all fields of scientific literature (18). According to the study design, almost two-thirds of the uncited articles were expert opinions, reviews, or case reports. This type of study was also prevalent among poorly cited articles. The large number of reviews is surprising given that this study design is common among top-cited articles. As reported by others, this study design accounts for 25% of the top-cited articles in implant dentistry (19), 19% in the dental literature (8), and 18% in orthodontics (20). The presence of a high number of case reports in both uncited (21.76%) and poorly cited articles (22.55%) is an interesting finding and confirms what other authors have highlighted in the field of oral and maxillofacial surgery (21). It should be noted that Case Reports’ tendency to receive few citations can affect the journal impact factor, and in the last decade, many editors have decided to no longer publish this type of article in their journals (22). However, a low citation rate does not necessarily indicate a small readership. Practitioners generally value case reports, an easy-to-understand scientific material (23). For instance, the Journal of Clinical Orthodontics, despite having a low impact factor, is one of the most popular journals among orthodontics practitioners and primarily publishes case reports. According to a recent bibliometric paper in the field of orthodontics, 46.6% of the published articles refer to an observational design, while 27.5% are translational; these two study designs are the most frequent (5). The same trend was observed in our poorly cited article cohort, probably because the greater the number of articles, the greater the possibility of being uncited. The lack of systematic reviews and RCTs in the uncited sample and the anecdotal presence of these study designs in the poorly cited sample suggest that these designs are more likely to receive a high number of citations. These findings are consistent with what has been reported in the field of maxillofacial surgery (24). In terms of study topics, among the uncited articles Litigation and legislation and Ethics and management and marketing accounted for 34.73% of the articles. First, we should note that some of these articles, particularly those belonging to the AJODO, could be considered editorials. However, as they were classified as articles on the same journal website, we decided to keep them in the articles pool. These topics are of great interest to readers since Orthodontic stakeholders understand how marketing and management are essential for practice success (25). Orthodontic providers also know how they must take great care to avoid and deal with malpractice litigation (26). Nevertheless, as reported by other authors, nonclinical studies or reports tend to be less cited because despite their interest, they contribute less to further research with a consequentially lower citation rate (9,27), which is possibly the reason behind the lower citation rate of such topics. It is remarkable that a topic such as skeletal anchorage, which, according to other authors, was among the most cited in the studied decade (6,20), is one of the most prevalent in the poorly cited articles. According to the study by Aura *et al*. (2019), the same topic was also the most prevalent among the whole articles production, in the same time span, involving 7.2% of the published articles, which demonstrates once again that if more publications are released on the same topic, there is also a greater risk of redundancy, which can lead to a lower citation rate. The same applies to topics such as Therapeutics, techniques, and procedures in second phase of treatment, Facial growth and development,; facial biotype, facial morphology, and dental arches; and chephalometry, radiology, CBCT, and scanners, which are almost equally prevalent in the poorly cited cohort as in the whole production of the analysed journals during the same time span. According to what was reported by Aura *et al*., who studied almost the same journals in the same decade, 5.1% of the articles were authored by a single author, 42.2% were authored by five or more authors, and 39% of the authors in his sample were female. This suggests that uncited and poorly cited articles are generally authored by fewer than average authors and that male authors are usually more prevalent.

## Conclusions

Based on the findings of the present study, the impact factor of a journal displays a strong negative correlation with the number of uncited or poorly cited articles. The occurrence of uncited and poorly cited articles is greater in orthodontics than in other medical disciplines. Topics related to litigation, legislation, ethics, and marketing tend to receive a low citation rate, which highlights that despite reader interest, the citation rate is low for subjects who are not clinical or cannot serve as a basis for the development of future research and therefore can be cited in subsequent publications. Furthermore, expert opinions and narrative reviews are common among uncited articles, while case reports are prevalent in both uncited and poorly cited articles.

## Figures and Tables

**Table 1:** The table shows the total number of articles (N) published by each journal (cited according to the NLM Title Abbreviation) in the years included in the study, the number of uncited articles (0), and the impact factor of each journal according to the SJCR (SJR). In the last column, the number of uncited articles is given as a percentage (0%) of the total number of articles published during the decade and the mean SJR during the study period (Mean/SJR).

		Am J of Orthod Dentofacial Orthop	Eur J Orthod	J Orofac Orthop	Aus orthod J	Korean J Orthod	Orthod Craniofac Res	Prog Orthod	Angle orthod	Semin Orthod	J Orthod	Total
2009	N	286	97	34	25	37	38	20	145	26	34	742
	0	7	0	0	0	2	1	2	0	0	2	14
	SJR	1.18	0.81	0.78	0.21	0.16	0.63	0.3	1	0.25	0.35	
2010	N	306	119	36	32	40	6	22	185	33	30	809
	0	9	2	1	3	5	1	1	2	2	3	29
	SJR	1.25	0.94	0.68	0.35	0.38	0.98	0.33	0.97	0.4	0.29	
2011	N	325	112	38	26	41	28	24	154	35	27	810
	0	13	0	2	3	6	0	0	0	7	3	34
	SJR	1.24	0.77	0.85	0.29	0.33	0.71	0.19	1.08	0.49	0.29	
2012	N	209	125	39	27	40	24	38	154	30	31	717
	0	13	0	0	4	0	2	4	0	1	3	27
	SJR	1.47	0.97	0.8	0.35	0.64	1.25	0.25	1.08	0.46	0.64	
2013	N	209	115	39	26	36	25	52	148	27	46	723
	0	14	0	1	3	0	0	0	0	3	3	24
	SJR	1.71	1.15	0.71	0.36	0.33	1.3	0.6	1.43	0.39	0.7	
2014	N	173	108	39	26	46	26	44	146	25	43	676
	0	2	0	3	4	0	0	0	1	2	1	13
	SJR	1.23	1.11	0.58	0.49	0.64	1.24	0.44	1.16	0.47	0.36	
2015	N	186	79	39	27	34	53	45	139	32	32	666
	0	3	0	1	2	0	0	0	0	0	8	14
	SJR	1.31	1.12	0.61	0.35	1	0.88	0.87	1.33	0.26	0.54	
2016	N	193	87	48	28	42	25	41	136	39	41	680
	0	0	2	5	6	0	0	0	0	15	5	33
	SJR	1.26	1.13	0.61	0.29	0.85	0.96	1.35	1.21	0.29	0.57	
2017	N	189	88	47		43	68	44	113	34	47	673
	0	2	0	2	-	0	0	0	1	5	5	15
	SJR	1.28	1.27	0.57	0.34	1.34	1.31	0.97	1.26	0.31	0.67	
2018	N	170	84	37	-	44	32	46	101	41	48	603
	0	3	1	1	-	1	0	0	1	2	4	13
	SJR	1.15	1.05	0.62	0.33	1.05	0.69	0.86	1.24	0.43	0.44	
Total	N	2246	1014	396	217	403	325	376	1421	322	379	7099
	0	66	5	16	25	14	4	7	5	37	37	216
		2.94%	0.49%	4.04%	11.52%	3.47%	1.23%	1.86%	0.35%	11.49%	9.76%	3.04%
	Mean/ SJR	1.31	1.03	0.68	0.34	0.67	1.00	0.62	1.18	0.38	0.49	

**Table 2:** Summary statistics of the included articles stratified by study design. The number (N) of articles per study design and the percentage (%) related to the total number of items in the specific cohort, uncited or poorly cited are presented.

Study Design					
Uncited articles			Poorly cited articles (1-3)		
Study design	N	%	Study design	N	%
Expert opinion	61	28.24	Observational	201	29.43
Case Report	47	21.76	Observational Cross-sectional	69	10.10
Narrative Review	46	21.30	Observational case-series	57	8.35
Observational	35	16.20	Observational case-control	52	7.61
Observational Cross-sectional	16	7.41	Observational Cohort	23	3.37
Observational case-series	11	5.09	Translational	179	26.21
Observational case-control	7	3.24	Translational Theorethical/models	95	13.91
Observational Cohort	1	0.46	Translational-human	30	4.39
Translational	24	11.11	Translational-animal	54	7.91
Translational Theorethical/models	11	5.09	Case Report	154	22.55
Translational-human	8	3.70	Narrative Review	95	13.91
Translational-animal	5	2.31	Expert opinion	22	3.22
Randomised controlled trial	2	0.93	Basic	18	2.64
Basic-Cells	1	0.46	Basic Materials	13	1.90
Total	216		Basic-Cells	5	0.73
			Randomised controlled trial	10	1.46
			Non Randomised controlled trial	2	0.29
			Systematic review	2	0.29
			Total	683	

**Table 3:** Summary statistics of the included articles stratified by article topic. The number (N) of articles per topic and the percentage (%) related to the total number of items in the specific cohort, uncited or poorly cited, are presented.

Uncited articles			Poorly cited articles (1-3)		
Topic	N	%	Topic	N	%
Litigation and Legislation and Ethics	53	24.54	Eruption problems: impaction, canine ectopic eruption/number problems (supernumeraries and agenesis)	44	6.44
Marketing and Management	22	10.19	Therapeutics, techniques, and procedures in second phase of treatment	43	6.30
Class II treatment	18	8.33	Bone anchorage: Implants and screws	40	5.86
Eruption problems: impaction, canine ectopic eruption/number problems (supernumeraries and agenesis)	11	5.09	Chephalometry, radiology, CBCT, scanners	39	5.71
Skeletal asymmetries/orthognathic surgery, corticotomies, bone distraction	11	5.09	Syndromes and systemic diseases, cystic and tumoral pathology	38	5.56
Stability and relapse/retention/fixed and removable retainers	9	4.17	Biomechanics—bone and periodontal biology during tooth movement	35	5.12
Chephalometry, radiology, CBCT, scanners	9	4.17	Facial growth and development, facial biotype, facial morphology, and dental arches	32	4.69
Materials	7	3.24	Bonding and bracket removal	31	4.54
Brackets design, friction, self-ligating	6	2.78	Skeletal asymmetries/orthognathic surgery, corticotomies, bone distraction	30	4.39
Class III treatment	5	2.31	Psychological and psychosocial aspects in patients: perception of esthetics, pain, comfort, quality of life, need of orthodontic treatment, collaboration	26	3.81
Biology	5	2.31	Retention and Stability	22	3.22
Injuries and complications during orthodontic treatment: periodontal, mucosal, nervous	5	2.31	Class II fixed or removable functional appliances	22	3.22
Bone anchorage: implants and screws	4	1.85	Skeletal Class III treatment (surgery not included)	17	2.49
Maxillary expansion	4	1.85	Genetics, hormones, chemical transmitters, medications	15	2.20
TMJ and craniomandibular dysfunction. Bruxism.	4	1.85	Maxilary expansion	14	2.05
History of orthodontics	3	1.39	Indexes and measurements/mathematical models, dental cast analysis	14	2.05
Aesthetic and perception of Aesthetics	3	1.39	Lip and cleft palate	14	2.05
Biomechanics	3	1.39	Orthodontic research	13	1.90
Facial growth and development, facial biotype, facial morphology, and dental arches	3	1.39	Management and marketing	12	1.76
Orthodontic education and training	3	1.39	Archwires, resins, and other materials: biochemistry, biology, toxicity	12	1.76
Social professional aspects,professional opinions	3	1.39	Litigation, legislation and ethic	12	1.76
Caries and decalcification	2	0.93	Class II treatment	11	1.61
Root resorbtion	2	0.93	Vertical alterations: open bite	11	1.61
Dental transplant	2	0.93	Upper airways, sleep apnea, snoring	11	1.61
Orthodontic research	3	1.39	TMJ and craniomandibular dysfunction. Bruxism.	11	1.61
Extraction and nonextraction therapy	2	0.93	Brackets design, friction, self-ligating	10	1.46
Others	14	6.48	Multidisciplinary treatment	10	1.46
Total	216		Periodontics	9	1.32
			Extraction and nonextraction therapy	9	1.32
			Injuries and complications during orthodontic treatment: periodontal, mucosal, nervous	8	1.17
			Cariology, traumatology/dental sensitivity/caries prevention: cariogenic microbiology, dental brushing	8	1.17
			Orthodontic education and certifications	8	1.17
			Mastication and malocclusion—posturology	7	1.02
			Root resorption and secondary defects during orthodontic treatment: dehiscence, recessions	6	0.88
			Materials	5	0.73
			Malocclusion etiology: etiologic factors, habits	5	0.73
			Dental Transplant	5	0.73
			Others	24	3.51
			Total	683	

**Table 4:** Summary statistics of Author-based and Article-based parameters. The number (N) of articles per topic and the percentage (%) related to the total number of items in the specific cohort, uncited or poorly cited, are presented.

Uncited articles			Poorly cited articles (1-3)		
	N	%		N	%
Authors			Authors		
1	113	52.31	1	92	13.47
2	30	13.89	2	96	14.06
3	21	9.72	3	124	18.16
4	25	11.57	4	136	19.91
5	13	6.02	5	107	15.67
> 5	14	6.48	> 5	128	18.74
Mean	2.31 ± 1.78		Mean	3.82 ± 1.98	
Male	362		Male	1808	
Female	137		Female	807	
M/F ratio	2.64		M/F ratio	2.24	
Institutions	166		Institutions	160	
University	142	85.54	University	512	91.76
Non Academic	24	14.46	Non Academic	46	8.24
North america	82	39.61	North america	185	26.54
Europe	67	32.37	Europe	226	32.42
Central and south america	7	3.38	Central and south america	15	2.15
Asia	27	13.04	Asia	207	29.70
Middle east	17	8.21	Middle east	44	6.31
Oceania	7	3.38	Oceania	15	2.15
			Africa	5	0.72
Funded	6	2.78	Funded	67	9.81
Unfunded	210	97.22	Unfunded	616	90.19
Open access	20	9.26	Open access	231	33.82
Subscription	196	90.74	Subscription	452	66.18

**Table 5:** The table shows the total number of articles (N) published by each journal (cited according to the NLM Title Abbreviation)in the years included in the study, the number of poorly cited articles (1-3), and the impact factor of each journal according to the SJCR (SJR). In the last column, the number of poorly cited articles is given as a percentage (1-3%) of the total number of articles published during the decade and the mean SJR during the study period (Mean/SJR).

		Am J of Orthod Dentofacial Orthop	Eur J Orthod	J Orofac Orthop	Aus orthod J	Korean J Orthod	Orthod Craniofac Res	Prog Orthod	Angle orthod	Semin Orthod	J Orthod	Total
2009	N	286	97	34	25	37	38	20	145	26	34	742
	1-3	16	3	1	7	17	0	8	2	2	6	62
	SJR	1.18	0.81	0.78	0.21	0.16	0.63	0.3	1	0.25	0.35	
2010	N	306	119	36	32	40	6	22	185	33	30	809
	1-3	12	7	3	6	17	3	4	10	7	2	71
	SJR	1.25	0.94	0.68	0.35	0.38	0.98	0.33	0.97	0.4	0.29	
2011	N	325	112	38	26	41	28	24	154	35	27	810
	1-3	13	6	4	4	13	2	6	4	8	2	64	
	SJR	1.24	0.77	0.85	0.29	0.33	0.71	0.19	1.08	0.49	0.29	
2012	N	209	125	39	27	40	24	38	154	30	31	717
	1-3	14	11	4	5	5	2	5	10	8	5	69
	SJR	1.47	0.97	0.8	0.35	0.64	1.25	0.25	1.08	0.46	0.64	
2013	N	209	115	39	26	36	25	52	148	27	46	723
	1-3	18	3	8	5	5	2	4	5	5	8	63
	SJR	1.71	1.15	0.71	0.36	0.33	1.3	0.6	1.43	0.39	0.7	
2014	N	173	108	39	26	46	26	44	146	25	43	676
	1-3	5	9	6	8	4	4	0	2	7	8	53
	SJR	1.23	1.11	0.58	0.49	0.64	1.24	0.44	1.16	0.47	0.36	
2015	N	186	79	39	27	34	53	45	139	32	32	666
	1-3	12	7	7	10	5	3	1	7	8	4	65
	SJR	1.31	1.12	0.61	0.35	1	0.88	0.87	1.33	0.26	0.54	
2016	N	193	87	48	28	42	25	41	136	39	41	680
	1-3	11	7	5	10	5	5	3	7	9	9	72
	SJR	1.26	1.13	0.61	0.29	0.85	0.96	1.35	1.21	0.29	0.57	
2017	N	189	88	47		43	68	44	113	34	47	673
	1-3	17	11	6		6	8	3	10	16	9	87
	SJR	1.28	1.27	0.57	0.34	1.34	1.31	0.97	1.26	0.31	0.67	
2018	N	170	84	37		44	32	46	101	41	48	603
	1-3	15	4	6		12	4	4	10	13	14	82
	SJR	1.15	1.05	0.62	0.33	1.05	0.69	0.86	1.24	0.43	0.44	
Total	N	2246	1014	396	217	403	325	376	1421	322	379	7099
	1-3	133	68	50	55	89	33	38	67	83	67	683
	1-3%	5.92%	6.80%	12.88%	26.27%	22.08%	10.46%	10.11%	4.64%	26.09%	17.68%	9.69%
	Mean/SJR	1.31	1.03	0.68	0.34	0.67	1.00	0.62	1.18	0.38	0.49	

## Data Availability

The datasets used and/or analyzed during the current study are available from the corresponding author.
